# Co-production of eENHANCE: a web platform to deliver personalised nutrition and exercise support to prevent cancer-related malnutrition and muscle loss

**DOI:** 10.1007/s00520-026-11428-w

**Published:** 2026-11-05

**Authors:** Nicole Kiss, Robin M. Daly, Lara Edbrooke, Linda Denehy, Jenelle Loeliger, Brenton Baguley, Natalie Maxwell Davis, Nicki Colls, Eric O., Annie Curtis, Patricia M. Livingston, Victoria White, Emily McNeil, Maddie Adair, Ailish Doyle, Anna Ugalde

**Affiliations:** 1 Institute for Physical Activity and Nutrition (IPAN), School of Exercise and Nutrition Sciences, Deakin University, Geelong, Australia; 2 Nutrition and Speech Pathology Department, Peter MacCallum Cancer Centre, Melbourne, Australia; 3 Physiotherapy Department, University of Melbourne, Parkville, Australia; 4 Centre for Health Services Research in Cancer, Peter MacCallum Cancer Centre, Parkville, Australia; 5 Sir Peter MacCallum Department of Oncology, University of Melbourne, Parkville, Australia; 6 Consumer Representative, Independent, Melbourne, Victoria Australia; 7 Faculty of Health, Deakin University, Geelong, Victoria Australia; 8 Centre for Quality and Patient Safety Research, School of Nursing and Midwifery, Institute for Health Transformation, Deakin University, Geelong, Victoria Australia; 9 School of Psychology, Faculty of Health, Deakin University, Geelong, Victoria Australia; 10 School of Exercise and Nutrition Sciences, Deakin University, Geelong, VIC Australia

**Keywords:** Nutrition, Exercise, Personalised, Self-guided, Digital health, Cancer

## Abstract

**Purpose:**

Access to nutrition and exercise services for people with cancer-related malnutrition varies across health services. This study co-produced a personalised self-guided evidence-based nutrition and exercise web program and evaluated feasibility and acceptability within clinical practice.

**Methods:**

A co-production approach was utilised following the four phases of Design Thinking in Healthcare. Phase 1 gathered user needs through interviews with consumers and health professionals, which were analysed using thematic analysis. Solutions were generated in Phase 2 to inform content and development of the platform. Phase 3 involved user acceptance testing. Feasibility testing in a clinical setting was undertaken in Phase 4.

**Results:**

Phase 1: interviews with 14 health professionals (100% female, mean age 59.5 [range 25–57] years) and five people with cancer (100% female, mean age 53.5 [range 57–62] years) generated three themes: (1) patient characteristics, (2) features and functions of the web platform and (3) optimising practice. Phase 2: an 8-week evidence-based self-guided nutrition and exercise program (eENHANCE) was developed based on the interview findings and refined in Phase 3 testing. Phase 4: 20 people with cancer participated in feasibility testing of eENHANCE at a tertiary cancer centre, with high (86%) acceptability of the intervention, compliance with study outcome assessment (67–100%), while 30–100% accessed prescribed modules.

**Conclusions:**

eENHANCE is a unique self-guided personalised nutrition and exercise program for people at risk of cancer-related malnutrition and muscle loss. High acceptability and feasibility indicate there may be potential to expand access to self-guided programs for people recovering from cancer treatment; however, further work is required in people undergoing treatment and to identify the most appropriate target groups and the optimal strategies to reach them to improve intervention fidelity.

**Supplementary information:**

The online version contains supplementary material available at https://doi.org/10.1007/s00520-026-11428-w.

## Introduction

Globally, there were almost 20 million new cases of cancer diagnosed in 2022 [1]. Cancer-related malnutrition and muscle loss may develop secondary to treatment toxicities, alterations in nutrient metabolism or absorption, or inflammation and affect one in three people undergoing cancer, which can increase to up to 60% in certain high-risk cancers such as lung and head and neck cancers [2]. Cancer-related malnutrition and muscle loss are associated with serious adverse consequences, including reduced survival, reduced ability to complete cancer treatment and an increased risk of life-threatening treatment toxicities [2–5]. However, randomised controlled trials have demonstrated the effectiveness of individualised nutrition and exercise interventions on improving survival and physical function and reducing treatment toxicities in people with cancer [6–9].

Several international guidelines and position statements recommend all people with cancer should have access to the core components of treatment for cancer-related malnutrition and muscle loss, including individualised nutrition therapy and appropriate exercise prescription [10–12]. However, access to nutrition and exercise services varies widely across health services, with people in regional and rural areas disproportionately affected and disadvantaged by these inequities [13–15]. Surveys of oncology health professionals consistently identify a lack of services to refer patients at risk of malnutrition or muscle loss and a lack of resources to screen and refer those in need as the top barriers to providing optimal evidence-based care [16, 17]. A study of health services in Australia has suggested that up to 50% of malnourished people with cancer are not under the care of a dietitian [18].

People with cancer and their carers describe nutrition as important throughout cancer treatment and recovery, although they also report limited support from health professionals regarding their nutritional needs [19]. Studies in people with advanced cancer reveal physical and nutritional needs are common, with up to 80% reporting unmet physical needs and almost 50% unmet nutritional needs [20, 21]. As allied health resources are already stretched within acute health services, innovative solutions to improve access to evidence-based care during treatment and recovery are required.

Digital technology enables wide-reaching interventions that can enhance the limited nutrition and exercise interventions currently offered in health services, meeting high demand without requiring additional staffing costs. A systematic review by our group found that technology-supported self-guided nutrition and physical activity interventions were safe and effective in improving physical activity level, dietary behaviour and fatigue in adults with cancer [22]. However, most of the technology-supported interventions in the systematic review were focused on healthy eating and physical activity after cancer treatment, with only two interventions for people undergoing active cancer treatment and none was designed to prevent or treat malnutrition and muscle loss throughout treatment and recovery [22]. Therefore, the aims of this study were as follows: (1) to co-produce a web-based platform (eENHANCE) with health professionals and consumers for personalised self-guided evidence-based nutrition and exercise support for people with cancer; (2) to test the feasibility, acceptability and fidelity of the web-based intervention in clinical practice and explore the effect on nutritional status, functional status, health-related quality of life and physical activity level.

## Methods

A co-production approach to consumer and health professional engagement was utilised for this study since the problem (lack of access to care) and solution (self-guided care) were understood, but optimal implementation needed to be established [23]. Co-production followed the steps described in Design Thinking in Healthcare: (1) understanding needs, (2) generating solutions, (3) prototyping and (4) testing [24].

### Steering committee

A steering committee was established at commencement of the project, comprising two consumer representatives, six clinical and research health professionals (three dietitians, two physiotherapists, one psychologist), two health services researchers, an exercise scientist and a senior research technology consultant. The steering committee provided input into the study protocol for each phase, contributed to the development of eENHANCE content, iterative development of the structure and functioning of eENHANCE and reviewed progress of the feasibility testing.

## Phase 1: Understanding the needs of end-users (June–November 2021)

### Methodological approach

The eENHANCE platform is intended to compliment usual clinical practice at health services where access to nutrition and exercise intervention does not align with clinical practice guidelines due to resource limitations. It is therefore crucial that the platform meets the needs of both the health service and patients who will use it. Through semi-structured interviews with health professionals, we sought insight into the features and functionality of eENHANCE that would augment current clinical care for the prevention or management of cancer-related malnutrition and muscle loss. Eligibility criteria for health professionals included dietitians, exercise physiologists and physiotherapists working with people with cancer within acute health services. The views of people with cancer were also sought through semi-structured interviews to understand their needs regarding features and functionality of eENHANCE that would augment current clinical care for the prevention or treatment of cancer-related malnutrition and muscle loss. Eligibility criteria for this group were people 18 years or older, currently undergoing treatment for cancer or completed cancer treatment within the previous 12 months and who experienced unintentional weight loss or reduced food intake during treatment. The semi-structured interview guide (Supplementary Table 1) aimed to understand participants’ perceptions of the critical components for engagement with a web-based platform, such as chatbot, text, videos, photos, reminders, tailored messages, links to external resources and animation, to deliver evidence-based nutrition and physical activity advice. Health professionals were also asked questions to inform the design of eENHANCE, taking into consideration how it could be implemented in practice. The interview guide was developed by the research team, who consist of people with lived experience and researchers, clinicians and health service managers with expertise in cancer, nutrition, exercise, psychology, behaviour change, implementation science, digital health and health services research. Interview questions were informed by the Capability, Opportunity, Motivation-Behaviour (COM-B) framework, an individual-level behaviour change framework involving three essential conditions: capability, opportunity and motivation for volitional behaviours [25].

### Recruitment

Health professionals were recruited through flyers distributed through professional organisations, including Dietitians Australia, the Victorian Cancer Malnutrition Collaborative, Exercise and Sports Science Australia, Australian Physiotherapy Association and alumni of the Masters of Clinical Exercise Physiology and Masters of Dietetics programs at Deakin University, as well as professional contacts of the research team. People with cancer were recruited through flyers distributed via the consumer networks of Cancer Council Victoria and Pancare Foundation. People interested in participating contacted a member of the research team, and a time was arranged for the interviews which were conducted online using Zoom video communications. Participants provided written consent and were emailed a short demographic questionnaire to complete prior to the interview.

### Analysis

Thematic analysis was undertaken to determine common needs and concepts. Interview audio recordings were transcribed verbatim, and transcripts were read by one author, a female dietitian (EM), who completed initial open coding. For rigour, the initial codes were reviewed by two authors (NK, AU), a female clinician researcher and female health services researcher who are trained and experienced in qualitative research, and regular meetings were held between EM, AU and NK throughout analysis to establish agreement regarding codes and to group codes into themes. Analysis was iterative and reflective to support understanding of needs and concepts and was conducted concurrently with data collection for identification of theoretical sufficiency [26].

## Phase 2: Generating solutions (November 2021–December 2022)

Themes identified in the semi-structured interviews informed the development of eENHANCE. Themes were presented to the steering committee, and a prototype of the platform structure and functionality was iteratively developed with the steering committee through a series of meetings and email correspondence to determine the number and topics of modules within the platform. The eENHANCE platform was subsequently developed by a senior research technology consultant. As module content was produced, it was circulated via email to steering committee members for feedback and iterative development.

## Phase 3: User acceptance testing of the prototype (January–April 2023)

Alpha testing, which involves assessing the input and output functions of the platform, was completed by the senior research technology consultant. This was followed by user acceptance testing with people with cancer, health professionals and members of the steering committee to assess the appropriateness of the design, ease of navigation and appearance. People with cancer and health professionals who participated in Phase 1 were invited to participate in the user acceptance testing. Participants were asked questions on the registration process, time to complete certain tasks, ease of navigation through the content and the readability and understandability of the modules and were invited to suggest content that they felt was missing and improvements to the platform. Responses to the questions were collated and informed any modifications to the eENHANCE platform in consultation with the steering committee.

## Phase 4: Pilot feasibility testing eENHANCE in clinical practice (April 2023 – November 2024)

### Setting and participants

Patients in the final week of radiotherapy for lower gastrointestinal, urological or breast cancer or who were within 2 weeks pre- or post-completion of their final chemotherapy cycle for any cancer type were recruited at Peter MacCallum Cancer Centre, a tertiary cancer centre in Melbourne, Australia. Patients were eligible if they were 18 years of age or older, not currently receiving or ineligible for dietetic care, able to ambulate independently, had a life expectancy of more than 6 months, Eastern Cooperative Oncology Group performance status ≤ 2, had access to an internet-connected device and had English language proficiency sufficient to understand the eENHANCE content and complete the patient-reported outcomes. Patients were excluded if they had a cancer or treatment type at high risk of requiring enteral or parenteral nutrition or high likelihood of automatic referral to a dietitian, severe or unstable neurological, cardiorespiratory or musculoskeletal condition that could compromise their ability to exercise safely, or an unstable psychiatric or cognitive disorder. The target sample size was 20 participants based on pragmatic considerations and recommended sample sizes for feasibility studies [27]. Eligible patients were identified from radiotherapy and chemotherapy patient lists and all participants provided written, informed consent. Participants were provided with access to eENHANCE for an 8-week period.

### Outcome assessment

Outcome measures and timing of assessment are described in Supplementary Table 2. Demographic, medical and social data were recorded from the medical record at baseline.

#### Feasibility

Rate of recruitment, attrition and compliance with completion of the study assessments were examined at the end of the study.

#### Intervention fidelity

System usage data were examined using the Deakin University built ADUCI user monitor and tracking system to determine participant engagement with the e-ENHANCE modules.

#### Acceptability

The Theoretical Framework of Acceptability (TFA) generic questionnaire was used to assess acceptability at 8 weeks, following completion of the e-ENHANCE intervention. The TFA assesses seven constructs of acceptability including (1) affective attitude (how an individual feels about an intervention), (2) burden (perceived effort to participate in the intervention), (3) ethicality (extent to which the intervention is a good fit with individual values), (4) intervention coherence (understanding of the intervention), (5) opportunity costs (benefits or values given up to engage in the intervention), (6) perceived effectiveness (perception of intervention achieving its purpose) and (7) self-efficacy (confidence in performing the behaviour to participate in the intervention) [28, 29]. In addition, semi-structured interviews with five participants explored experiences of the study procedures and intervention to supplement the quantitative TFA (Supplementary Table 3). Participants were approached to take part in an interview once they completed the intervention, and interviews were limited to the first five who consented to minimise the burden of participation. Interviews were recorded and transcribed verbatim using Zoom Video Communications with transcripts checked for accuracy by one author (ARC) and content analysis completed in NVivo (version 15).

Weight, nutritional status and body composition were assessed by a trained dietitian using standard protocols: Weight (kg) was measured using standard floor scales (Tanita InnerScan 50, Tanita Australia). Nutritional status was assessed using the Patient Generated Subjective Global Assessment (PG-SGA) [30]. Fat-free mass (FFM, kg) was measured using tetrapolar bioimpedance spectroscopy (BIS) using a commercially available device (Sozo, Impedimed) and normalised for height to generate the FFM index (FFMI).

Physical function and muscle strength were assessed by a trained physiotherapist using standard protocols: Physical function was assessed using the 30 s sit-to-stand test (30STS), a validated measure to assess lower limb strength and physical function [31]. Handgrip strength dynamometry (Jamar™) was used to test upper limb muscle strength, which was tested bilaterally, three times following a practice trial with the maximum strength (kg) recorded from either hand used.

#### Dietary intake

Dietary intake was assessed from 3-day food records, one of the recommended dietary assessments by the National Cancer Institute for prospective observational studies [32], to determine average daily energy (kJ) and protein (g) intake. Dietary data were imported into ASA24 for analysis.

#### Physical activity

Self-reported physical activity (metabolic equivalent tasks [MET]-min/week) was assessed using the International Physical Activity Questionnaire – Short Form (IPAQ-SF) [33]. Objective physical activity (sedentary time [minutes], step count, light and moderate intensity activity time [minutes] was measured over a 7-day period using an accelerometer, the valid and reliable Actigraph GT3XP and considered valid when a minimum of 8 hours per day for 4 days wear time was recorded [34].

#### Health-related quality of life

The health-related quality of life was measured using the Functional Assessment of Cancer Therapy – General (FACT-G), a self-reported instrument validated for use in people with cancer [35]. The FACT-G consists of 27 core items to assess patient function in four domains: physical, social/family, emotional and functional well-being.

#### Safety

Adverse events, defined as any undesirable event, such as injury, fall and discomfort related to the intervention were recorded and the severity reported according to the Common Terminology Criteria for Adverse Events version 5 [36].

### Statistical analysis

Descriptive statistics were used to summarise demographic, clinical characteristics, feasibility and acceptability outcomes and exploratory outcomes at baseline and follow-up. Patient-reported outcome measures were scored according to questionnaire guidelines. Mean difference and 95% confidence intervals from baseline to follow-up were summarised for clinical outcomes. Data were analysed using Stata 18 (StataCorp, TX, USA).

Ethics approval was obtained for all phases of work, from Deakin University Health Ethics Advisory Group (HEAG-H 48_2021) for Phases 1 to 3 and from Peter MacCallum Cancer Centre Human Research Ethics Committee (HREC/94206/PMCC) for Phase 4. Participants provided written informed consent for involvement in all phases. The study was conducted in accordance with the Declaration of Helsinki [37].

## Results

### Phase 1: Understanding the needs of end users

Interviews were completed with 14 health professionals (mean [range] age 59.5 [25–57] years, 100% female, 64% dietitians, 36% physiotherapists) and five people living with cancer (mean [range] age 53.5 [57–62] years, 100% female, 40% residing in regional locations). The mean duration of interviews was 28 min (range 17–46 min). Three themes were identified: (1) patient characteristics, (2) features and functions of the web platform and (3) optimising practice. The three themes and their categories are presented in Supplementary Table 4 with supporting quotes from health professionals [HP1 to HP14] and people with cancer [PT1 to PT5]. In theme one, health professionals noted the need to consider patient characteristics with regard to accessing the platform, particularly the potential for variation in health and technology literacy, motivation and the impact of their current health status. In theme two, the features deemed important by health professionals and people with cancer included simple messages, incorporating user-friendly images and video, reminders and rewards, comprehensive and credible information, personalised content and easy navigation. Theme three was related to considerations for future implementation of eENHANCE in practice, with clinician awareness and endorsement of eENHANCE, sustainability, flexibility in how it is used and variation in service delivery across healthcare settings identified as important factors.

### Phase 2: Generating solutions

**Fig. 1:**
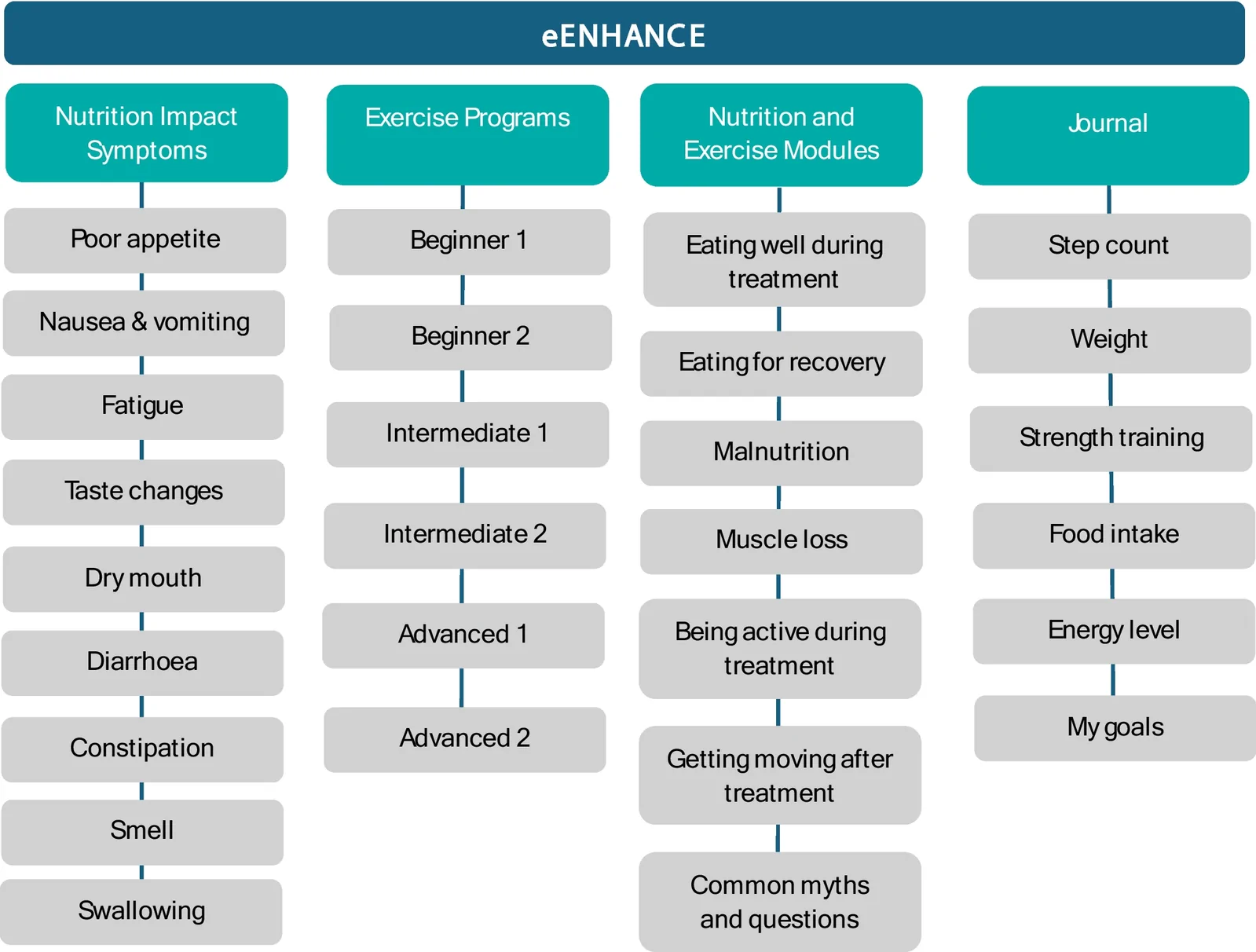
Modules available within the eENHANCE program

The eENHANCE platform is designed to provide an 8-week individualised nutrition and exercise program to people at risk of cancer-related malnutrition and muscle loss. eENHANCE contains fixed modules which are accessible to users at any time during the 8-week program as well as dynamic modules that are personalised to individual users based on self-identified needs (Fig. 1). Upon registration, users are presented with a short questionnaire based on the validated Patient-Generated Subjective Global Assessment Short Form (PG-SGA-SF) [30] to identify weight changes, the presence of symptoms impacting nutritional intake and questions regarding current level of engagement (if any) with resistance-based training. Following completion of the questionnaire, an algorithm provides users with access to modules tailored to their self-identified symptoms and nutritional concerns and an exercise program tailored to their current ability (Supplementary Fig. 1). A weekly reminder email is sent to users with a link to complete the same questionnaire to ensure the personalised content remains responsive to changing self-identified needs. The content is evidence-based, developed by expert clinicians and researchers and includes written material, video animations and links to evidence-based resources to cater to different levels of health literacy, with the video animations representing the audiovisual version of the written content. Behaviour change techniques utilised in eENHANCE were derived from barriers and facilitators identified from the phase 1 qualitative interviews. The key techniques identified included goal setting, progress journalling, motivational reminders and strategies to overcome barriers to eating well. The techniques used align with Goals and Planning (e.g. goal setting, action planning, problem solving), Feedback and Monitoring (self-monitoring) and Antecedents (prompts/cues) in Mitchie et al.’s Behaviour Change Technique Taxonomy v1 [38]. eENHANCE is adaptive to smartphones, tablets and desktop computers providing flexibility for users. The eENHANCE intervention is flexible and can be used with as little or as much health professional involvement as staffing resources permit within individual health services.

### Phase 3: User acceptance testing of the prototype

User acceptance testing was completed by two consumers, one health professional and one researcher (3 [75%] females, mean age 48.5 [34–63] years) who were also involved in phase 1. Testing was undertaken on a mobile device (*n* = 3, 75%) or desktop computer (*n* = 1, 25%). All participants ‘agreed’ or ‘strongly agreed’ that registration was straightforward, the content was engaging and the exercise program instructions were easy to follow. The overall impression of the platform was ‘positive’ or ‘very positive’ for all participants. Areas identified as needing improvement were how to navigate the platform and find content, simpler language in the written content and larger font size. In response, an instructional video was embedded in the platform to guide users in navigating the content and features, and the written content was revised with simpler language and larger font.

### Phase 4: Pilot feasibility testing in clinical practice

#### Feasibility

**Fig. 2:**
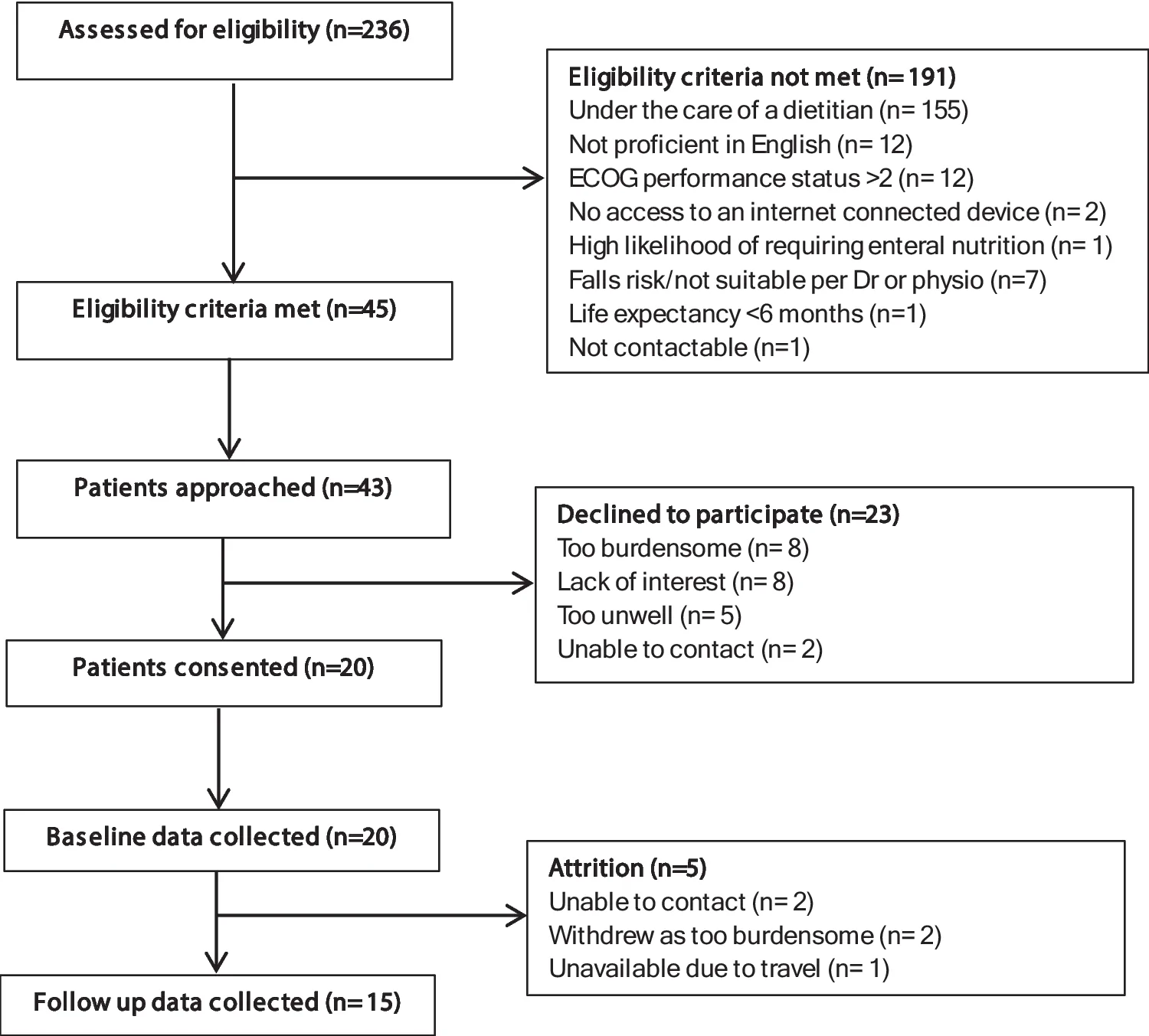
Participant flow chart

Of the 236 patients screened, 45 (19%) met the eligibility criteria, 43 (96%) were approached to participate and 20 (46%) agreed to participate (Fig. 2). Characteristics of the 20 participants are described in Table 1. The most common reasons for declining to participate were ‘study assessments too burdensome’ (*n* = 8) or ‘lack of interest’ (*n* = 8). Over the duration of the 8-week intervention, three (15%) participants were lost to follow-up and two (10%) participants withdrew due to the burden of participation. Compliance with study assessments was 100% (*n* = 20/20) at baseline for all assessments except for accelerometer wear (*n* = 17/20, 85%) and dietary intake (*n* = 16/20, 80%). Compliance at follow-up was also 100% (*n* = 15/15) for all assessments apart from dietary intake (*n* = 11/15, 73%) and accelerometer wear (*n* = 10/15, 67%).

**Table 1:** Phase 4 participant characteristics

Characteristic	*N* (%)
Age (years) mean (SD)	49.3 (11.3)
Female	17 (85)
Living situation	
Alone	4 (20)
With others	16 (80)
Education	
Secondary	8 (40)
Tertiary	12 (60)
Cancer diagnosis	
Breast	17 (85)
Prostate	2 (10)
Colorectal	1 (5)
Treatment	
Radiotherapy	19 (95)
Chemotherapy	1 (5)

#### Intervention fidelity

All participants completed the baseline questionnaire and were prescribed a personalised program. Participants logged into eENHANCE a mean (SD) of 8.7 (12.3) times during the 8-week intervention and spent a median total time of 39 min (range 0 to 623) using the modules. However, participants could download their exercise program to engage with the content offline, which is not reflected in this total time. Of the fixed modules which were accessible to all participants at any time during the intervention, the modules accessed by the highest proportion of participants were ‘Getting moving after treatment’ (*n* = 10, 50%), ‘Common myths and questions’ (*n* = 10, 50%) and ‘Eating for recovery’ (*n* = 9, 45%) (Supplementary Table 5). The fixed modules that were accessed the highest number of times during the intervention were ‘Eating for recovery’ (*n* = 33 times), ‘Getting moving after treatment’ (*n* = 31 times) and ‘Being active during treatment’ (*n* = 23 times). Of the fixed modules prescribed to participants based on self-identified needs, 30% to 100% of participants accessed their prescribed content (Supplementary Table 5). Most participants (*n* = 12, 60%) were prescribed a beginner exercise program and 5 (42%) accessed the program, while 80% (*n* = 4) and 100% (*n* = 3) of those prescribed an intermediate or advanced exercise program accessed it (Supplementary Table 5). The journal was accessed by 3 (15%) participants.

#### Acceptability

The TFA questionnaire was completed by 14 (70%) participants. In total, 86% reported being ‘comfortable’ or ‘very comfortable’ and ‘confident’ or ‘very confident’ using eENHANCE. The effort required to use the platform varied, as did participants’ clarity regarding how it would help manage their risk of malnutrition or muscle loss and the perception of whether it interfered with other priorities. Overall, 86% of participants rated eENHANCE as ‘acceptable’ or ‘very acceptable’ (Table 2). Participant interviews found a positive response to eENHANCE, with most finding the program easy to use and navigate, useful for their needs and all indicated they would recommend the program to other people with cancer (Supplementary Table 6).

**Table 2:** Responses to the theoretical framework of acceptability questionnaire (*n*= 14)

TFA construct	Questionnaire item					
Affective attitude	How comfortable did you feel using the e-ENHANCE platform?	**Very uncomfortable**	**Uncomfortable**	**No opinion**	**Comfortable**	**Very comfortable**
		0 (0)	0 (0)	2 (14%)	7 (50%)	5 (36%)
Burden	How much effort did it take to use the e-ENHANCE platform?	**No effort at all**	**A little effort**	**No opinion**	**A lot of effort**	**Huge effort**
		0 (0)	10 (71%)	1 (8%)	3 (21%)	0 (0%)
Ethicality	How well does the e-ENHANCE platform fit with the way you would like to manage your risk of cancer-related malnutrition or muscle loss?	**Not a very good fit**	**Not a good fit**	**No opinion**	**A good fit**	**A very good fit**
		0 (0)	2 (14%)	2 (14%)	5 (36%)	5 (36%)
Perceived effectiveness	The e-ENHANCE platform has improved my ability to manage my risk of cancer-related malnutrition or muscle loss	**Strongly disagree**	**Disagree**	**No opinion**	**Agree**	**Strongly agree**
		1 (8%)	3 (21%)	1 (8%)	6 (42%)	3 (21%)
Intervention coherence	It is clear to me how e-ENHANCE will help manage my risk of cancer-related malnutrition or muscle loss	**Strongly disagree**	**Disagree**	**No opinion**	**Agree**	**Strongly agree**
		1 (8%)	1 (8%)	1 (8%)	7 (50%)	4 (26%)
Self-efficacy	How confident do you feel about using the e-ENHANCE platform?	**Very unconfident**	**Unconfident**	**No opinion**	**Confident**	**Very confident**
		0 (0)	0 (0)	2 (14%)	7 (50%)	5 (36%)
Opportunity costs	Using the e-ENHANCE platform interfered with my other priorities	**Strongly disagree**	**Disagree**	**No opinion**	**Agree**	**Strongly agree**
		2 (14%)	5 (36%)	3 (21%)	3 (21%)	1 (8%)
General acceptability	How acceptable was the e-ENHANCE platform to you?	**Completely unacceptable**	**Unacceptable**	**No opinion**	**Acceptable**	**Completely acceptable**
		0 (0)	0 (0)	2 (14%)	8 (60%)	4 (26%)

#### Clinical outcomes

A summary of clinical outcomes at baseline and follow-up is reported in Supplementary Table 7. At baseline 25% (*n *= 5) of participants were malnourished according to PG-SGA. Overall, 75% of participants improved or maintained their PG-SGA category and 70% improved or maintained their IPAQ-SF physical activity level (Supplementary Table 7). There was a high proportion of missing data for dietary intake and objectively measured accelerometer physical activity, with 50% and 55% of participants missing data at one or both timepoints. Therefore, results for these clinical outcomes may not be an accurate representation.

## Discussion

This study describes the development of a personalised self-guided web-based intervention, eENHANCE, for the prevention or treatment of cancer-related malnutrition or muscle loss and the evaluation of the feasibility and acceptability of eENHANCE within clinical practice. The eENHANCE platform was developed to address the issue of inequitable access to evidence-based nutrition and exercise services for people with cancer across health services [2, 14, 39]. eENHANCE provides evidence-based nutrition and exercise support tailored to individual needs and is accessible to anyone through an internet connected device.

The use of the Design Thinking in Healthcare co-production approach to consumer and health professional engagement [23, 24] in the development of eENHANCE ensured end user needs were addressed from the outset. In our interviews with people with cancer and health professionals to inform the development of eENHANCE, an ability to accommodate varying levels of health literacy, ensuring the credibility of information, easy navigation, the use of reminders and rewards and safety considerations were deemed important and included in the final platform. These features, in particular ease of navigation, weekly reminders and the motivational aspects of eENHANCE, were viewed favourably by participants in our feasibility testing study suggesting their relevance to patients. Similar features have been deemed important by codesign participants in previous studies of codesigned health applications [40].

Although considered important by consumers and health professionals during development, opportunities for patient connection and functionality to link patients and health professionals were not included due to budget constraints and will be revisited in future iterations of the platform. Feedback from participants during the feasibility testing, also indicated interest in opportunities for connectiveness through virtual classes or groups with these features raised as areas where improvements could be made to eENHANCE. A previous study has reported challenges with including interactive features, such as discussion boards, within an app for carers of people with cancer due to the potential to affect usability for people less confident in using technology [41]. However, interactive components are clearly important to people using digital interventions and should be considered in future iterations of the eENHANCE platform, potentially utilising closed domain artificial intelligence that draws from a curated, peer-reviewed knowledge base to reduce but not eliminate the need for health professional oversight. Future iterations of eENHANCE would benefit from iterative engagement of stakeholders, including consumers, health professionals and health service managers in refining the platform and how it could be integrated into health care [42]. In particular, further representation of male participation is required to address their limited participation in the work thus far, with consideration of recruitment strategies to attract a broad and diverse range of participants.

A high proportion of patients who met the eligibility criteria were approached to participate in the feasibility study and of those approached, approximately half agreed to participate with 25% attrition at follow-up. This indicates further exploration is required to understand the patients who would engage with and benefit most from this intervention and consider effective engagement strategies, particularly for those who perceive the intervention as burdensome yet are likely to benefit. Initially, the eligibility criteria required patients to have been screened as at risk of malnutrition and not eligible to access dietetic care. However, the feasibility study was conducted in a relatively well-resourced tertiary health service with established malnutrition screening processes where patients at risk of malnutrition are prioritised for dietetic care, although they may not all receive exercise care. During eligibility screening, it became apparent that most patients at risk of malnutrition were eligible for dietetic care, and therefore, this criterion was removed. This resulted in eENHANCE being tested with participants who may not have been at risk of malnutrition, and indeed, only 25% of participants in the feasibility study were malnourished. This in turn may have influenced interest in participating, perceived acceptability of the intervention as well as the modules participants engaged with during the 8-week feasibility testing. There was high compliance with the initial study assessments and slightly lower compliance at follow-up which was reflective of the 25% attrition and predominantly due to the need to attend in person for the assessment. We observed lower compliance for completion of the dietary assessment and accelerometer wear due to the additional burden of completing these assessments, which is consistent with previous research [43]. Tracking data revealed varying levels of engagement with the prescribed content in the platform among participants. Approximately half of participants utilised modules that contained information relevant for the recovery period, reflecting the post-treatment status of the study participants. A small proportion of participants utilised the journal feature of eENHANCE and the reasons for this warrant further investigation and consideration in future iterations of the platform.

Overall, the eENHANCE platform was highly acceptable, with most participants in the feasibility study reporting that they were comfortable using the platform and post-intervention interviews demonstrated an overwhelmingly positive perception of eENHANCE. Of note, 28% of participants reported they disagreed that the eENHANCE platform had improved their ability to manage their risk of cancer-related malnutrition and muscle loss, although only 16% felt that it was not clear how the eENHANCE platform would help manage their risk. Although the overall acceptability was high, findings for these nuanced constructs of acceptability may differ in patient populations with a higher risk of malnutrition. Interestingly, although the study was not designed for statistical inference in terms of effectiveness, we observed clinically important trends in maintenance or improvement in nutritional status and self-reported physical activity, suggesting participants may be benefiting from the eENHANCE intervention. It is also possible this was attributable to natural recovery following the completion of treatment. However, it is well established that treatment side effects and decline in nutritional and functional status can persist in 6 to 8 weeks following completion of cancer treatment [10].

Consideration of incorporating the additional features and functionality suggested throughout the phases of the current study should be undertaken. Following further refinement of eENHANCE, a randomised trial is required in a broader group of patients who are known to be at risk of cancer-related malnutrition or muscle loss. Future work needs to determine the ability of this self-guided individualised digital intervention to improve patient outcomes, including nutritional status, muscle mass, physical function and treatment completion and the most appropriate duration of the intervention including whether this should be individualised based on need. Considering that eENHANCE may be suitable for people living with cancer who want to receive nutrition and exercise interventions yet are unable to access these services through their treating healthcare provider, further insight is needed from key stakeholders regarding how eENHANCE could be integrated into the healthcare system to reach these people. In view of the increasing need for self-management options within health services, this would inform future risk stratification, implementation and scalability of the intervention should it demonstrate clinical benefits.

A limitation of this study was the small number of consumers, all female, who participated in phase 1, which sought to understand the needs of end users. This may have limited the generalisability of the needs identified in this phase [44], and further insights from a broader range of consumers and other stakeholders will be incorporated into future work and iterations of eENHANCE. A further limitation was the feasibility testing of eENHANCE in a relatively well-resourced tertiary cancer centre with an established malnutrition screening, referral and treatment system in place. In addition, the feasibility study participants were mostly female patients with breast cancer limiting generalisability [44]. During the feasibility study, data were not collected on whether participants were currently working or the presence of comorbidities which may have affected engagement with eENHANCE.

In conclusion, eENHANCE is an innovative digital platform to support self-guided personalised nutrition and exercise intervention for people living with cancer and at risk of cancer-related malnutrition and muscle loss. The co-production approach likely contributed to the high acceptability of eENHANCE and suggests potential to improve access to nutrition and exercise interventions for people recovering from cancer treatment in health services where limited resources are available. High acceptability alongside indications of clinically important benefits suggests that a larger trial is needed to determine clinical and cost effectiveness of the intervention and to examine the characteristics of patients who are more likely to engage with and derive benefit from this type of intervention. However, further refinement of the eENHANCE is first required to incorporate participant feedback. In addition, exploration of optimal integration of eENHANCE into the healthcare system and engagement with health professionals in community and primary care settings is required to reach people who are most likely to engage with and benefit from the intervention prior to implementation and scaling.

## Supplementary information

Below is the link to the electronic supplementary material.

ESM 1 (DOCX 81.7 KB)

## Data Availability

The data generated and analysed during this study are available from the corresponding author on reasonable request.

## References

[1] Bray F, Laversanne M, Sung H, Ferlay J, Siegel RL, Soerjomataram I, Jemal A (2022) Global cancer statistics 2022: GLOBOCAN estimates of incidence and mortality worldwide for 36 cancers in 185 countries. CA Cancer J Clin 74(3):229–263 10.3322/caac.21834 38572751

[2] Marshall KM, Loeliger J, Nolte L, Kelaart A, Kiss NK (2019) Prevalence of malnutrition and impact on clinical outcomes in cancer services: a comparison of two time points. Clin Nutr 38(2):644–651 10.1016/j.clnu.2018.04.007 29789167

[3] Blauwhoff-Buskermolen S, Versteeg KS, Schueren MAEdvd, Braver NRd, Berkhof J, Langius JAE, Verheul HMW (2016) Loss of muscle mass during chemotherapy is predictive for poor survival of patients with metastatic colorectal cancer. J Clin Oncol 34(12):1339–1344 10.1200/JCO.2015.63.6043 26903572

[4] Prado CMM, Baracos VE, McCargar LJ, Reiman T, Mourtzakis M, Tonkin K et al (2009) Sarcopenia as a determinant of chemotherapy toxicity and time to tumor progression in metastatic breast cancer patients receiving capecitabine treatment. Clin Cancer Res 15(8):2920–2926 10.1158/1078-0432.CCR-08-2242 19351764

[5] Prado CMM, Lieffers JR, McCargar LJ, Reiman T, Sawyer MB, Martin L, Baracos VE (2008) Prevalence and clinical implications of sarcopenic obesity in patients with solid tumours of the respiratory and gastrointestinal tracts: a population-based study. Lancet Oncol 9(7):629–635 10.1016/S1470-2045(08)70153-0 18539529

[6] Cormie P, Zopf EM, Zhang X, Schmitz KH (2017) The impact of exercise on cancer mortality, recurrence, and treatment-related adverse effects. Epidemiol Rev 39(1):71–92 10.1093/epirev/mxx007 28453622

[7] McTiernan A, Friedenreich CM, Katzmarzyk PT, Powell KE, Macko R, Buchner D et al (2019) Physical activity in cancer prevention and survival: a systematic review. Med Sci Sports Exerc 51(6):1252–1261 10.1249/MSS.0000000000001937 PMC6527123 31095082

[8] Schuetz P, Fehr R, Baechli V, Geiser M, Deiss M, Gomes F et al (2019) Individualised nutritional support in medical inpatients at nutritional risk: a randomised clinical trial. Lancet 393(10188):2312–2321 10.1016/S0140-6736(18)32776-4 31030981

[9] van der Werf A, Langius JAE, Beeker A, ten Tije AJ, Vulink AJ, Haringhuizen A et al (2020) The effect of nutritional counseling on muscle mass and treatment outcome in patients with metastatic colorectal cancer undergoing chemotherapy: a randomized controlled trial. Clin Nutr 39(10):3005–3013 10.1016/j.clnu.2020.01.009 32037284

[10] Arends J, Bachmann P, Baracos V, Barthelemy N, Bertz H, Bozzetti F et al (2017) ESPEN guidelines on nutrition in cancer patients. Clin Nutr 36(1):11–48 10.1016/j.clnu.2016.07.015 27637832

[11] Hayes SC, Newton RU, Spence RR, Galvão DA (2019) The Exercise and Sports Science Australia position statement: exercise medicine in cancer management. J Sci Med Sport 22(11):1175–1199 10.1016/j.jsams.2019.05.003 31277921

[12] Kiss N, Loeliger J, Findlay M, Isenring E, Baguley BJ, Boltong A et al (2020) Clinical Oncology Society of Australia: position statement on cancer-related malnutrition and sarcopenia. Nutr Diet 77(4):416–425 10.1111/1747-0080.12631 PMC7540290 32803904

[13] Baguley BJ, Benna-Doyle S, Drake S, Curtis A, Stewart J, Loeliger J (2024) Access to nutrition services and information after active cancer treatment: a mixed methods study. J Cancer Surviv 18(1):176–185 10.1007/s11764-023-01352-x PMC10866769 36823493

[14] Lopez E, Banbury M, Isenring E, Marshall S (2019) Malnutrition point prevalence from 2012 to 2019 and assocaited outcomes in adult patients in rural hopsitals. J Ageing Research Clin Practice 8:91–97

[15] Shipton DA, Dennett AM, Sayner AM, Tang CY (2022) A national survey of oncology physiotherapy services for cancer survivors in Australia. Asia Pac J Clin Oncol 18(5):e404–e413 10.1111/ajco.13739 35098664

[16] Deftereos I, Kiss N, Brown T, Carey S, Carter VM, Usatoff V et al (2021) Awareness and perceptions of nutrition support in upper gastrointestinal cancer surgery: a national survey of multidisciplinary clinicians. Clin Nutr ESPEN 46:343–349 10.1016/j.clnesp.2021.09.734 34857218

[17] Kiss N, Bauer J, Boltong A, Brown T, Isenring L, Loeliger J et al (2020) Awareness, perceptions and practices regarding cancer-related malnutrition and sarcopenia: a survey of cancer clinicians. Support Care Cancer 28(11):5263–5270 10.1007/s00520-020-05371-7 32103357

[18] Marshall K LJ (2012) Investigating practices relating to malnutrition in Victorian Cancer Services - a summary report. Department of Health: State Government of Victoria

[19] Loeliger J, Dewar S, Kiss N, Drosdowsky A, Stewart J (2021) Patient and carer experiences of nutrition in cancer care: a mixed-methods study. Support Care Cancer. 10.1007/s00520-021-06111-1 33710413

[20] Choi KH, Park JH, Park SM (2011) Cancer patients’ informational needs on health promotion and related factors: a multi-institutional, cross-sectional study in Korea. Support Care Cancer 19(10):1495–1504 10.1007/s00520-010-0973-z 20694821

[21] Hwang SS, Chang VT, Cogswell J, Alejandro Y, Osenenko P, Morales E et al (2004) Study of unmet needs in symptomatic veterans with advanced cancer: incidence, independent predictors and unmet needs outcome model. J Pain Symptom Manage 28(5):421–432 10.1016/j.jpainsymman.2004.02.023 15504619

[22] Kiss N, Baguley BJ, Ball K, Daly RM, Fraser SF, Granger CL, Ugalde A (2019) Technology-supported self-guided nutrition and physical activity interventions for adults with cancer: systematic review. JMIR Mhealth Uhealth 7(2):e12281 10.2196/12281 PMC6390188 30747720

[23] Vargas C, Whelan J, Brimblecombe J, Allender S (2022) Co-creation, co-design, co-production for public health - a perspective on definition and distinctions. Public Health Res Pract. 10.17061/phrp3222211 35702744

[24] Altman M, Huang T, Breland J (2018) Design thinking in healthcare: systematic review. Preventing Chronic Disease Public Health Researcg, Practice and Policy, p 15 10.5888/pcd15.180128 PMC6178900 30264690

[25] Michie S, van Stralen MM, West R (2011) The behaviour change wheel: a new method for characterising and designing behaviour change interventions. Implement Sci 6(1):42 10.1186/1748-5908-6-42 PMC3096582 21513547

[26] Braun V, Clarke V (2021) To saturate or not to saturate? Questioning data saturation as a useful concept for thematic analysis and sample-size rationales. Qual Res Sport Exerc Health 13(2):201–216

[27] Whitehead AL, Julious SA, Cooper CL, Campbell MJ (2016) Estimating the sample size for a pilot randomised trial to minimise the overall trial sample size for the external pilot and main trial for a continuous outcome variable. Stat Methods Med Res 25(3):1057–1073 10.1177/0962280215588241 PMC4876429 26092476

[28] Sekhon M, Cartwright M, Francis JJ (2017) Acceptability of healthcare interventions: an overview of reviews and development of a theoretical framework. BMC Health Serv Res 17(1):88 10.1186/s12913-017-2031-8 PMC5267473 28126032

[29] Sekhon M, Cartwright M, Francis JJ (2022) Development of a theory-informed questionnaire to assess the acceptability of healthcare interventions. BMC Health Serv Res 22(1):279 10.1186/s12913-022-07577-3 PMC8887649 35232455

[30] Ottery F (1996) Definition of standardized nutritional assessment and interventional pathways in oncology. Nutrition 12(1, Supplement):S15–S19 10.1016/0899-9007(96)90011-8 8850213

[31] Bowman A, Denehy L, Benjemaa A, Crowe J, Bruns E, Hall T et al (2022) Feasibility and safety of the 30-second sit-to-stand test delivered via telehealth: an observational study. PM R. 10.1002/pmrj.12783 35138036

[32] National Cancer Institute. Dietary assessment primer: national cancer institute. Available from: https://dietassessmentprimer.cancer.gov/profiles/table.html. Accessed 19 May 2026

[33] Craig CL, Marshall AL, Sjöström M, Bauman AE, Booth ML, Ainsworth BE et al (2003) International Physical Activity Questionnaire: 12-country reliability and validity. Med Sci Sports Exerc 35(8):1381–1395 10.1249/01.MSS.0000078924.61453.FB 12900694

[34] Demeyer H, Burtin C, Van Remoortel H, Hornikx M, Langer D, Decramer M et al (2014) Standardizing the analysis of physical activity in patients with COPD following a pulmonary rehabilitation program. Chest 146(2):318–327 10.1378/chest.13-1968 PMC4122275 24603844

[35] Cella DF, Tulsky DS, Gray G, Sarafian B, Linn E, Bonomi A et al (1993) The Functional Assessment of Cancer Therapy scale: development and validation of the general measure. J Clin Oncol 11(3):570–579 10.1200/JCO.1993.11.3.570 8445433

[36] National Cancer Institute (2017) Common terminology criteria for adverse events (CTCAE) version 5.0. Available from: https://ctep.cancer.gov/protocoldevelopment/electronic_applications/ctc.htm#ctc_50. Accessed 19 May 2026

[37] World Medical Association (2024) Declaration of helsinki: world medical association. Available from: https://www.wma.net/what-we-do/medical-ethics/declaration-of-helsinki/. Accessed 22 Sept 2026

[38] Michie S, Richardson M, Johnston M, Abraham C, Francis J, Hardeman W et al (2013) The behavior change technique taxonomy (v1) of 93 hierarchically clustered techniques: building an international consensus for the reporting of behavior change interventions. Ann Behav Med 46(1):81–95 10.1007/s12160-013-9486-6 23512568

[39] Dennett AM, Peiris CL, Shields N, Morgan D, Taylor NF (2017) Exercise therapy in oncology rehabilitation in Australia: a mixed-methods study. Asia Pac J Clin Oncol 13(5):e515–e527 10.1111/ajco.12642 28004526

[40] Al-Mondhiry J, D’Ambruoso S, Pietras C, Strouse T, Benzeevi D, Arevian AC, Wells KB (2022) Co-created mobile apps for palliative care using community-partnered participatory research: development and usability study. JMIR Form Res 6(6):e33849 10.2196/33849 PMC9264134 35737441

[41] Heynsbergh N, Heckel L, Botti M, O SC, Livingston PM (2019) Development of a smartphone app for informal carers of people with cancer: processes and learnings. JMIR Form Res 3(2):e10990 10.2196/10990 PMC6482398 30973346

[42] Kiss N, Jongebloed H, Baguley B, Marshall S, White VM, Livingston PM et al (2024) Meaningful consumer involvement in cancer care: a systematic review on co-design methods and processes. JNCI Cancer Spectr. 10.1093/jncics/pkae048 PMC11240760 38897655

[43] Kiss N, Prado CM, Abbott G, Edbrooke L, Denehy L, Curtis AR et al (2025) Sociodemographic and clinical factors associated with low muscle mass and composition in people treated with (chemo)radiotherapy for lung cancer. Eur J Clin Nutr 79(4):369–378 10.1038/s41430-024-01552-3 39614103

[44] Zhang J, Dong B, Dong H, Guo D, Yuan Y, Tian L (2025) Gender-specific differences in factors influencing supportive care needs in colorectal cancer patients. BMC Cancer 25(1):471 10.1186/s12885-025-13887-6 PMC11907775 40087644

