# Supplementary material for: Co-production of eENHANCE: a web platform to deliver personalised nutrition and exercise support to prevent cancer-related malnutrition and muscle loss
Source: Support Care Cancer. 2026 Nov 5;34(12):1197. doi: 10.1007/s00520-026-11428-w (PMC13740113; doi:10.1007/s00520-026-11428-w)
Supplement: Supplementary file 1 [file 520_2026_11428_MOESM1_ESM.docx]

**Supplementary Material: Co-production of eENHANCE: a web-platform to deliver personalised nutrition and exercise support to prevent cancer-related malnutrition and muscle loss**

Supplementary Figure 1: Overview of the structure and flow of the eENHANCE platform

Supplementary Table 1: Phase 1 semi-structured interview questions

**Health Professionals**

1. How do you feel about patients using a web-platform for nutrition and exercise advice?
   a. Prompts: confidence, support needed

(COM-B conditions: Capability, Motivation)

2. Are there any factors you think would affect their likelihood of using a web-platform for nutrition and exercise advice?
   a. Prompts: support/ encouragement from health professional, reminders, tailored messages, accessibility (via smart device)

(COM-B conditions: Opportunity, Motivation)

3. Are there any factors that could prevent them from using a web-platform for nutrition and exercise advice?
   a. Prompts: if so, can you suggest how these could be minimised?

(COM-B conditions: Capability, Opportunity, Motivation)

4. Are there any features of a web-platform that would increase the likelihood patients would engage with the content/ encourage you to do the interventions?
   a. Prompts: videos, instructions, chatbot, written content, content tailored to your needs, visual appeal (what does this mean to you), links to external resources?

(COM-B conditions: Capability, Opportunity, Motivation)

5. Are there any features of a web-platform that would reduce the likelihood they would engage with the content?
   a. Prompts: if so, can you suggest how these could be minimised, e.g. intuitive navigation?

(COM-B conditions: Capability, Opportunity, Motivation)

6. How do you think such a web-platform would integrate with current clinical practice?
   a. Prompts: degree of support, tailoring of content from treating health professional, self-guided use by patients

(COM-B conditions: Capability, Motivation)

**People with cancer**

1. How do you feel about using a web-platform for nutrition and exercise advice?
   a. Prompts: confidence, support needed

(COM-B conditions: Capability, Motivation)

2. Are there any factors that would affect your likelihood of using a web-platform for nutrition and exercise advice?
   a. Prompts: support/ encouragement from health professional, reminders, tailored messages, accessibility (via smart device)

(COM-B conditions: Opportunity, Motivation)

3. Are there any factors that could prevent you from using a web-platform for nutrition and exercise advice?
   a. Prompts: if so, can you suggest how these could be minimised?

(COM-B conditions: Capability, Opportunity, Motivation)

4. Are there any features of a web-platform that would increase the likelihood you would engage with the content/ encourage you to do the interventions?
   a. Prompts: videos, instructions, chatbot, written content, content tailored to your needs, visual appeal (what does this mean to you), links to external resources?

(COM-B conditions: Capability, Opportunity, Motivation)

5. Are there any features of a web-platform that would reduce the likelihood you would engage with the content?
   a. Prompts: if so, can you suggest how these could be minimised, e.g. intuitive navigation?

(COM-B conditions: Capability, Opportunity, Motivation)

6. How do you see such a web-platform fitting with nutrition and exercise care provided by a health professional?
   b. Prompts: degree of support or tailoring of content from treating health professional, self-guided use by patients

(COM-B conditions: Capability, Opportunity)

Supplementary Table 2: Outcome assessment measures and timing of assessments

| **Measure** | **VISIT 1**  **Baseline**  **(final week of radiotherapy; or within two weeks pre- or post-completion of final chemotherapy cycle)** | **e-ENHANCE intervention**  **8-weeks** | **VISIT 2**  **Follow up**  **Week 8** |
| --- | --- | --- | --- |
| Demographic, medical and social information | X |  |  |
| Feasibility (recruitment, attrition, compliance with study measures) |  |  | X |
| Acceptability survey |  |  | X |
| Weight and nutritional status | X |  | X |
| Body composition | X |  | X |
| Physical activity (self-reported and objective measures) | X |  | X |
| Functional status (30STS) | X |  | X |
| Dietary intake | X |  | X |
| Muscle strength | X |  | X |
| Health-related quality of life | X |  | X |
| Safety | <- -------------------------------------------------------------------------------------------> | | |

Supplementary Table 3: Phase 3 interview questions

**Study procedures and assessments**

1. Can you tell me what you thought of the information about the study you were provided when you were invited to participate in the study?
   a. Was it helpful?
   b. Was there anything missing that you would have liked to know?
   c. How do you think we could promote the study to others?

2. You were asked to complete a number of different questionnaires and other assessments at the beginning and end of the study. How did you feel about having to do these assessments?
   a. What about coming into Peter Mac for the assessment—how did you feel about that?

3. How did you feel about wearing the activity monitor and keeping the food diary during the study? Did you have any difficulties with that? Any advice on how we can improve this component of the study?

**e-ENHANCE intervention**

1. What did you think about the e-ENHANCE platform?
   a. Were there aspects you liked?
   b. Were there aspects you disliked?
   c. Was it easy to navigate?

2. Would you want to continue using it?

3. Who do you think would most benefit?
   a. Are there any people/groups you would not recommend this for?

4. Is there anything else you would like tell us about your experiences of taking part in the study or using e-ENHANCE?

5. Would you recommend e-ENHANCE to other people?
   a. Why or why not?

Supplementary Table 4: Summary of themes and supporting codes

| **Themes** | **Supporting codes** | **Supporting quotes** |
| --- | --- | --- |
| (1) Patient characteristics | Patient access to the platform | ‘…it would depend on obviously, the patient demographic.’ [HP 5]  ‘It depends on the age of the patient so more elderly patients would probably struggle.’ [HP 1] |
|  | Variations in health literacy | ‘From a health literacy perspective, we have some patients who can't really cope with a lot of written literature.’ [HP 4] |
|  | Patient understanding and ability to use technology | ‘I found that there is a percentage of people last year when I was doing telehealth appointments, that just couldn't manage the technology.’ [HP 8]  ‘I guess it's just whether, you know, they're comfortable with the internet as a whole.’ [HP 11] |
|  | Patient motivation | ‘I think it very much depends on the patient and very motivated patients will do everything that the clinician says and appreciate the extra support and take it on board.’ [HP 3]  ‘If the app had everything in it, like a package, like the first video showing you the benefits, you wouldn't specifically need much more encouragement. If, if it showed the absolute benefits, which there are.’ [PT 1] |
|  | Patient diagnosis and current health status | ‘I think a web-based programme would be helpful for patients that have to travel quite far or are quite frail.’ [HP 3]  ‘When I was really, really sick, I'm just holding the phone like this, like on the phone to Centerlink for nearly an hour, and that I had to go and lie down to hold the phone. And it was I was that weak.’ [PT 1] |
| (2) Features and functions of the web-platform | Simplifying messages through images and video | ‘Videos explaining concepts, rather than written content … I think that's always a lot more engaging for people.’ [HP 12]  ‘Videos would be amazing here, that sort of thing would be great. Yeah, just something that we're not already giving them.’ [HP 9] |
|  | Complimenting current care | ‘I think it would certainly be good for patients to have access to something for when they're not seeing a dietitian.’ [HP 7]  ‘It is a good addition to someone's clinical practice, but it shouldn't replace when you would normally be picking up the phone or getting them back face to face.’ [HP 12] |
|  | Accommodating varying patient literacy | ‘Having it pitched at a grade seven level or below, I think that's what they recommend for readability. That way you capture everyone, and you're not talking about some people's health literacy.’ [HP 1]  ‘If you give me a video on something, somebody talking, I can follow through a lot easier. Maybe because English is my second language. And there's a lot of people that you have to consider. The other is a lot of people from other countries as well.’ [PT 2] |
|  | Reminders, feedback and rewards | ‘If you messaged people, and they had something as a reminder, it would be helpful for them to have a look at it.’ [HP 13]  ‘There really is a thing called chemo fog, or chemo brain. And so those tools, like a reminder might be more essential, because there's so much else happening, but you can't expect that person to necessarily keep track of what they should be doing.’ [PT 3] |
|  | Engaging and interactive content | ‘Minimising how much information you give people. Because, if it's shorter and more catchy, they're more likely to look at it. If it's too much detail and too wordy they're much less likely to look at it.’ [HP 13]  ‘Finding ways to incorporate the right foods with easy recipes and things is really interesting. For me, I like cooking, and I like recipes. And I like food, obviously. So, I find that really good.’ [PT 5] |
|  | Opportunity for patient connection | ‘I know that peer support is a really good thing to have and oncology survivors can communicate with each other and know that they're not alone and share experiences.’ [HP 7]  ‘Lots of our patients, particularly a lot of the head and neck patients, and the breast cancer patients as well, that we see really value that peer support. I think there would be interest from some people to be able to kind of jump online with and share stories and that sort of thing.’ [HP 14] |
|  | Updating and monitoring | ‘It would have to kind of remain it would have to remain up to date with the most recent evidence.’ [HP 10]  ‘Making sure that all the information is up to date, and accurate.’ [HP 12] |
|  | Linking patients and health professionals | ‘I would have information that would be looking at red flags, if you're experiencing these symptoms, you need to see a health professional, speak to your doctor about this, or see a dietitian,’ [HP 8]  ‘I had to ask my doctor, my oncologist, if they had an oncology dietitian, but they didn't. And they didn't have any list of dietitians for me. They said, go to the Yellow Pages, which I thought was really sad. I asked at my hospital, they said, no, you can't access us outside the hospital.’ [PT 5] |
|  | Comprehensive and credible information | ‘If it was recommended by a kind of reputable organisation, like I know, a lot of the patients on our ward reference the Cancer Council or the leukaemia Foundation.’ [HP 10]  ‘If patients had access to some recipes or some sort of consistent messaging potentially, I mean I know all the hospitals have their own resources that they give patients for education. Well, just a list of places that the patients could go to for that sort of information, like different credible websites.’ [HP 2] |
|  | Ease of navigation of the platform | ‘I suppose if I found it really difficult to use, that would definitely prevent me from using it.’ [PT 4]  ‘It needs to be easy to use on all devices.’ [HP 6] |
|  | Consideration of safety | ‘If it had really clear guidelines on when it's safe or not to exercise, because that's definitely something that people worry about, that they shouldn't be exercising, because they’re sick or it's not safe. So, really clear guidelines to the patient on when it's okay, and when to stop.’ [HP 10]  ‘Maybe the videos of the exercises have different levels, beginners, somebody who's advanced to whatever benchmark is and then can move to the next level. So, you don't run the risk of someone incorrectly performing exercises or hurting themselves.’ [PT 3] |
|  | Personalised content | ‘Something more interactive than just pictures on a screen, or things, something a bit more that patient can get involved with, and it's a bit more tailored to the individual. [HP 5]  ‘It needs to be for different levels. If you had a 30 year old that went through the same thing, they would come out of it a lot different to a 50-60 year old coming out of the same treatment.’ [PT 1] |
| (3) Optimising practice | Clinician awareness and endorsement | You definitely have to have the medical team on board, especially with the older population, because they their trust is instilled in the doctor system.’ [HP 6]  ‘If an oncologist or staff from the day infusion centre, someone like that, if they said, oh, yes you seem to be struggling with your diet, our losing weight or nausea. Yeah, if they recommended it. Yeah, it probably would have been something I would look at.’ [PT 4] |
|  | Sustainable promotion | ‘Having like champions that are going to keep spruiking the message about the platform, it doesn't just get lost over time.’ [HP 11] |
|  | Opportunities to embed the platform in clinical practice | I think from a nursing staff screening perspective, I would expect a lot of services could put this [web-platform] in as something that, if someone is screened at the beginning of their treatment, or midway, being at risk, but kind of low risk, that they could be directed to the system information to kind of get them started’ [HP 14]  ‘If you are someone that can access health services, your health professional can always go through with you [the platform] or ask you to be at a certain stage of the web platform say if it was going through week by week, asking them to be up to week three, depending on where you're at.’ [PT 2] |
|  | Flexible care | ‘I think maybe a combination of sort of some online stuff that they might doing their own time versus some live things to be more engaged in.’ [HP 2]  ‘Could we do say week one, and then once they've completed that it goes straight on to say to week two? Or should it allow them a month to complete section one, and then go on, just to slow it down? And I guess not, not make it so overwhelming? So even if they have an interruption in their life, or they get sick, or they bounce back in hospital, even if it's three weeks later, they can still tap back in at week two.’ [HP 4] |
|  | Variations in health services | ‘Recognising each healthcare service as a unique healthcare service. And engaging the right people from that service.’ [HP 11]  ‘The one aspect of care that really lacks support here, and there is probably some serious risk of malnutrition is getting radiotherapy privately. There is no allied health there, there's no support, they don't do anything with it. They watch them lose weight, and they don't sort of refer on to anyone or have support services.’ [HP 9] |

Supplementary Table 5: Participant engagement with the content of eENHANCE from system usage data

| **Modules** | **Number of participants prescribed the module**  **N (%)** | **Number of participants prescribed the module who accessed the module**  **N (%)** | **Number of times the module was accessed during the 8-week intervention (total)**  **N** |
| --- | --- | --- | --- |
| **Nutrition impact symptom modules (dynamic modules)^a^** |  |  |  |
| Managing poor appetite | 2 (10) | 1 (50) | 2 |
| Managing nausea and vomiting | 2 (10) | 0 (0) | - |
| Managing fatigue | 10 (50) | 3 (30) | 6 |
| Coping with taste changes | 2 (10) | 1 (50) | 2 |
| Dealing with dry mouth | 2 (10) | 1 (50) | 1 |
| Managing diarrhoea | 2 (10) | 0 (0) | - |
| Managing constipation | 4 (20) | 2 (50) | 8 |
| Aversion to smell of food | 1 (5) | 1 (100) | 1 |
| Difficulty Swallowing | 0 (0) | - | - |
| **Nutrition and exercise modules (fixed modules)^b^** |  |  |  |
| Eating well during treatment | 20 (100) | 7 (35) | 20 |
| Eating for recovery | 20 (100) | 9 (45) | 33 |
| What is cancer-related malnutrition | 20 (100) | 6 (30) | 16 |
| What is cancer-related muscle loss | 20 (100) | 6 (30) | 11 |
| Being active during treatment | 20 (100) | 6 (30) | 23 |
| Getting moving after treatment | 20 (100) | 10 (50) | 31 |
| Common myths and questions | 20 (100) | 10 (50) | 13 |
| **Exercise program modules** |  |  |  |
| Beginner 1 | 12 (60) | 6 (50) | 52 |
| Beginner 2 | 0 (0) | - | - |
| Intermediate 1 | 5 (25) | 4 (80) | 38 |
| Intermediate 2 | 0 (0) | - | - |
| Advanced 1 | 3 (15) | 3 (100) | 15 |
| Advanced 2 | 0 (0) | - | - |

^a^Dynamic modules are prescribed to individual users based on self-identified needs; ^b^fixed modules are accessible to all users at any time during the intervention period.

Supplementary Table 6: Participant experience of the eENHANCE intervention (n= 5)

| **Component** | **Summary of responses** | **Example quotes** |
| --- | --- | --- |
| Navigation of eENHANCE | Most participants found eENHANCE easy to use and navigate. | “I would [recommend eENHANCE], because it's easy to use. You know, even when you're going through treatment, it's easy, even when you're sick, you can look at it and watch a few videos.” (PT9)  “I found it easy to use, I could easily use it on my phone.” (PT1) |
| Most useful aspects of eENHANCE | The most useful aspects of eENHANCE were different from participant-to-participant; however, all interviewed participants found at least one aspect (e.g., exercise videos or nutrition support) which was enjoyable and helped to improve aspects of their health or well-being. eENHANCE provided participants with motivation to focus on their own health and wellbeing. | “Because I'm not a fit person, I think I really liked the idea and the reminders and having a programme and it feels like your kind of not doing it on your own.” (PT10)  “I think [strength training] was the bit I found the most valuable of it all. So, I'm someone who really needs encouragement to do these sorts of things. And also, you don't really know what to do. I mean, it’s all very well saying you should do strength training but what exactly do you do? I mean, what sort of exercise are you to do? So that bit was really, really good.” (PT20)  “I felt rewarded when I'm not as puffed going upstairs and all of that stuff. So you sort of think okay, I can feel that I'm stronger and I’m getting better. And you know, even though I'm still sleeping a lot, I'm I am getting fitter.” (PT3)  “I really liked the modules around the diet. So, the information provided for the constipation in particular, that was the one that I found most useful. And I did see improvement in that aspect of my life.” (PT1)  “For someone like me, who's quite good at not focusing on themselves, it actually made me focus on myself and my diet, and not just being a mum and a carer and a wife and doing all that stuff.” (PT3)  “I think the programme is good for keeping it in my consciousness, like having those reminders and having exercise targeted at my level is good, like that makes it easier.” (PT10) |
| Opportunities to improve eENHANCE | Participants of eENHANCE provided several key suggestions to improve the usability of the platform, including the need for tailored support and regular automated reminders. The types of exercises provided in eENHANCE were not suitable to all participants, and this aspect of the platform may be improved by integrating peer support components. | “I think like maybe an improvement would be if it could send notifications to you, so it reminded you like, “have you got out and exercised today” or “have you done the program”, maybe something like that, just so it's in your face a bit more. Because it can be easy just to ignore it.” (PT1)  “I don't know whether you could set up reminders [to complete the exercises] at all in your online thing because I think that would be great.” (PT3)  “It'd be good if you could log on and have like a virtual class. So, if you had like a physio or PT that was taking the class, that could be cool. But yeah, other than that, I think that they are the standard exercises that you're used to doing at the gym, but that's just not for me. I prefer like HIIT classes or Pilates and things like that.” (PT 1)  “I think exercise in a way that's more fun, like maybe in a group would be more fun. Because to me the exercises, for me, they weren't fun.” (PT10) |
| Overall perception of eENHANCE | eENHANCE was well received by participants, and most participants would recommend the platform to other people with cancer, including participants of any age. | “I got a lot out of it. It made me feel more positive about what I'm going through. And that there are things that you can do to enhance your wellbeing and I think reduce the risk of the cancer coming back.” (PT9)  “I've had this sort of direct support for me for 10 weeks. So, I haven't felt like I needed a breast care nurse or go to my doctor very much to get extra advice. So, I think it was good, because it covered most of the symptoms that you get from treatment.” (PT3)  “I think everyone who's undergone treatment should have access to [the eENHANCE platform].” (PT20)  “Now that I'm eating better, in the last few weeks, I've gone back to participating in indoor soccer and also restarted back at the gym. So, I think [eENHANCE] did help me to get back to sort of that normal life path. So yeah, I'm glad that I participated in the study. (PT1) |

Supplementary Table 7: Clinical outcomes at baseline and follow-up visit

| **Outcome measure** | **Baseline visit**  **Mean (SD)**  **n (%)** | **8-week visit**  **Mean (SD)**  **n (%)** |
| --- | --- | --- |
| Weight (kg) | 68.2 (15.5) | 70.0 (17.6)^1^ |
| PG-SGA score | 4.8 (3.4) | 2.7 (1.5)^1^ |
| PG-SGA category  Well-nourished  Malnourished  Improved/maintained  Worsened  Missing data at follow up | 15 (75)  5 (25) | 15 (75)  0 (0)  5(25) |
| Fat-free mass index (kg/m^2^) | 16.1 (3.5) | 17.6 (4.8)^1^ |
| 30-second sit-to-stand | 15.1 (5.9) | 18.2 (7.4)^1^ |
| Maximum handgrip strength (kg) | 28.8 (7.5) | 30.1 (7.5)^1^ |
| FACT-G score | 78.2 (16.8) | 86.4 (15.8)^1^ |
| IPAQ-SF MET minutes | 1974.2 (1614.0) | 2913.7 (2588.7)^1^ |
| IPAQ-SF physical activity level  Low  Moderate  High  Improved/maintained  Worsened  Missing data at follow up | 5 (25)  8 (40)  7 (35) | 1 (5)  8 (40)  6 (30)  5 (25) |
| Energy intake (kJ)  Missing data at baseline or follow up | 8821.3 (3824.5)  4 (20) | 7777.3 (2164.9)  9 (45) |
| Protein intake (g)  Missing data at baseline or follow up | 91.8 (41.8)  4 (20) | 81.0 (18.8)  9 (45) |
| Average daily sedentary time (minutes)  Missing data at baseline or follow up | 1230.6 (73.1)  3 (15) | 1241.1 (58.7)  10 (50) |
| Average daily light activity (minutes)  Missing data at baseline or follow up | 170.4 (60.9)  3 (15) | 161.0 (51.5)  10 (50) |
| Average daily moderate to vigorous activity (minutes)  Missing data at baseline or follow up | 41.4 (18.9)  3 (15) | 37.9 (13.1)  10 (50) |
| Average daily step count  Missing data at baseline or follow up | 6271.1 (2745.9)  3 (15) | 6426.2 (1876.3)  10 (50) |

^1^Missing data for 5 participants at follow-up; PG-SGA, patient-generated subjective global assessment; FACT-G, functional assessment of cancer therapy – general, IPAQ-SF, international physical activity questionnaire short form; MET, metabolic equivalent time
